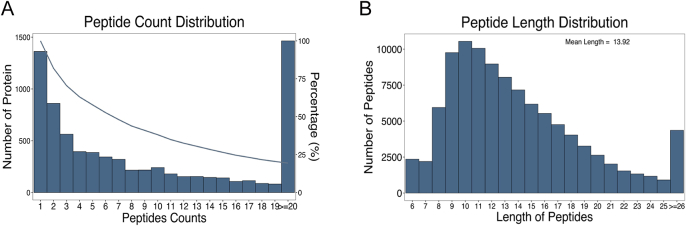# The viability and coagulation function of storing platelets can be maintained in plasma without cryoprotectants at −80 °C

**DOI:** 10.1016/j.rpth.2026.106635

**Published:** 2026-05-08

**Authors:** Xuan Dang, Erxiong Liu, Zhixin Liu, Mei Wang, Shunli Gu, Ning An, Yiqing Wang, Hanlu Fan, Xingbin Hu, Yaozhen Chen

**Affiliations:** Department of Transfusion Medicine, Xijing Hospital, Fourth Military Medical University, Xi’an, Shaanxi, China

**Keywords:** Blood coagulation, cryopreservation, blood platelets

## Abstract

**Background:**

To prolong the storage period of platelets, dimethyl sulfoxide (DMSO) has been used for platelet cryopreservation at −80 °C to prevent cold storage lesions. Nevertheless, it is important to note that DMSO is a toxic chemical agent that may cause adverse effects and potential safety concerns for clinical applications. Therefore, many studies have attempted to reduce the DMSO concentration used for platelet cryopreservation. Based on our previous findings regarding the stability of platelets in platelet-rich plasma stored at −80 °C for 1 month, it is imperative to explore the cryopreservation of platelets in plasma.

**Objectives:**

This study aimed to evaluate the feasibility of storing apheresis platelets in plasma at −80 °C as a supplemental solution in the absence of cryoprotectants, such as DMSO.

**Methods:**

We used a comprehensive approach, analyzing platelet count, morphology, mitochondrial structure, and metabolomic and proteomic profiles to assess storage lesions.

**Results:**

Our findings revealed that platelets stored at −80 °C in plasma maintained a stable cell count and procoagulant function, comparable with those stored with DMSO. Proteomic analysis showed that the fundamental protein composition essential for platelet clotting function was largely preserved, while metabolomic analysis suggested that metabolism had a minimal impact on platelet homeostasis. *In vitro* assessments showed no significant difference in platelet activation, and *in vivo* studies confirmed comparable bleeding times and platelet counts posttransfusion.

**Conclusion:**

These results demonstrate that storage in plasma at −80 °C effectively preserves platelet viability and functionality over a short period. This study highlights the potential for developing supplementary storage strategies as alternatives to conventional preservation methods, which could improve platelet availability in transfusion medicine.

## Essentials

• Dimethyl sulfoxide (DMSO) is used in –80 °C platelet storage despite its toxicity.
• This study evaluated platelet storage in plasma at –80 °C without DMSO.
• Platelets stored in plasma retained stable count, procoagulant function, and key proteins.
• It offers a short-term supplementary strategy for platelet preservation in transfusion medicine.

## 1 Introduction

Platelets play a crucial role in maintaining the balance between thrombosis and hemostasis in humans [1]. Consequently, platelet transfusion is required for numerous therapeutic interventions across a range of clinical conditions, including hematological disorders, trauma, surgery, and massive transfusion [[2], [3], [4]]. Unlike red blood cells, platelets are difficult to store *in vitro* while avoiding storage lesions and preserving functional integrity [[5], [6], [7]]. The currently accepted international standard for platelet storage recommends storage at 20 ± 2 °C with constant agitation [8]. However, the shelf life of platelets using this method is limited to only 5 to 7 days, which presents significant challenges for the management of this blood product. Furthermore, the risk of bacterial contamination remains a major safety concern for the clinical use of such stored platelets [9,10]. Although cold storage at 4 ± 2 °C was abandoned several decades ago, this strategy has been revisited in recent years. While this condition does extend the platelet shelf life, it remains limited to just 14 days [11,12]. In addition, platelets stored under cold conditions exhibit more rapid clearance *in vivo* than those stored at room temperature [13,14]. As a result, the hemostatic function of platelets stored under cold conditions lasts only a few hours posttransfusion, necessitating a greater supply of this blood component.

To prolong the storage period of platelets, dimethyl sulfoxide (DMSO) has been used for platelet cryopreservation at −80 °C to prevent cold storage lesions [15]. Platelets recovered using this method can maintain good viability and coagulation function after transfusion. Currently, a concentration of 5% to 6% DMSO is recommended as a cryoprotectant for cold storage [16]. France and Germany have approved the use of frozen platelets at −80 °C for military operations, while the Dutch Army has also implemented deep cryogenic preservation of platelets at this temperature in military settings. This development indicates that frozen platelets have gradually become an essential reserve blood resource in military contexts. Research conducted by Liu et al. [17] at the General Hospital of the People’s Liberation Army of China has demonstrated that platelet-rich plasma containing 5% DMSO can be stored at −80 °C for 1 to 16 months. However, it is important to note that DMSO is a toxic chemical substance that may induce protein degradation and potentially cause damage to human organs [[18], [19], [20]]. This issue has attracted considerable attention, and efforts to reduce the DMSO concentration are ongoing [21]. At present, there is a lack of relevant industry standards to support DMSO-based platelet cryopreservation in China, leading many blood banks to discontinue the supply of frozen platelet preparations. Considering our previous findings on the stability of platelets in platelet-rich plasma stored at −80 °C for 1 month [22], it is important to further investigate the potential of platelets preserved in plasma at this temperature, particularly in comparison with DMSO-preserved platelets.

In the current study, we compared platelets stored at −80 °C solely in plasma with those stored with DMSO. Following storage and thawing, we analyzed platelet concentration, apoptosis, morphology, metabolomics, and proteomics to evaluate storage lesions. Additionally, the functionality of platelets stored at −80 °C solely in plasma vs those stored with DMSO was assessed both *in vitro* and *in vivo*. Our findings indicate that apheresis platelets stored solely in plasma at −80 °C are well-preserved, suggesting they may serve as a suitable option for clinical use as a supplement in the absence of cryoprotectants.

## 2 Methods

### 2.1 Preparation and storage of platelets

Platelets were collected from 20 healthy volunteers meeting standard blood donation criteria using an apheresis device (Amicus 4R4580; Fenwal) with acid citrate dextrose-adenine as the anticoagulant at a whole blood-to-anticoagulant ratio of ∼1:9, as per standard guidelines. The platelet yield for apheresis was set to 4.0 × 10^11^ platelets per collection, suspended in a volume of ∼250 to 300 mL of plasma, in accordance with American Association of Blood Banks standards and the specifications of the apheresis device. The collection procedure was performed according to standard operating procedures without additional manual centrifugation steps, and the final platelets were stored in the autologous plasma of the donors. All collected units were leukoreduced, with residual white blood cell counts <5 × 10^6^ per unit as required by American Association of Blood Banks guidelines. Residual red blood cell contamination was negligible, as determined by visual inspection and automated cell counting (where applicable). The apheresis device operated with a whole blood flow rate maintained at 60 to 80 mL/min, using the manufacturer’s standard centrifugation protocols for platelet collection. The total procedure time ranged from 30 to 45 minutes per donor.

**Figure 1:**
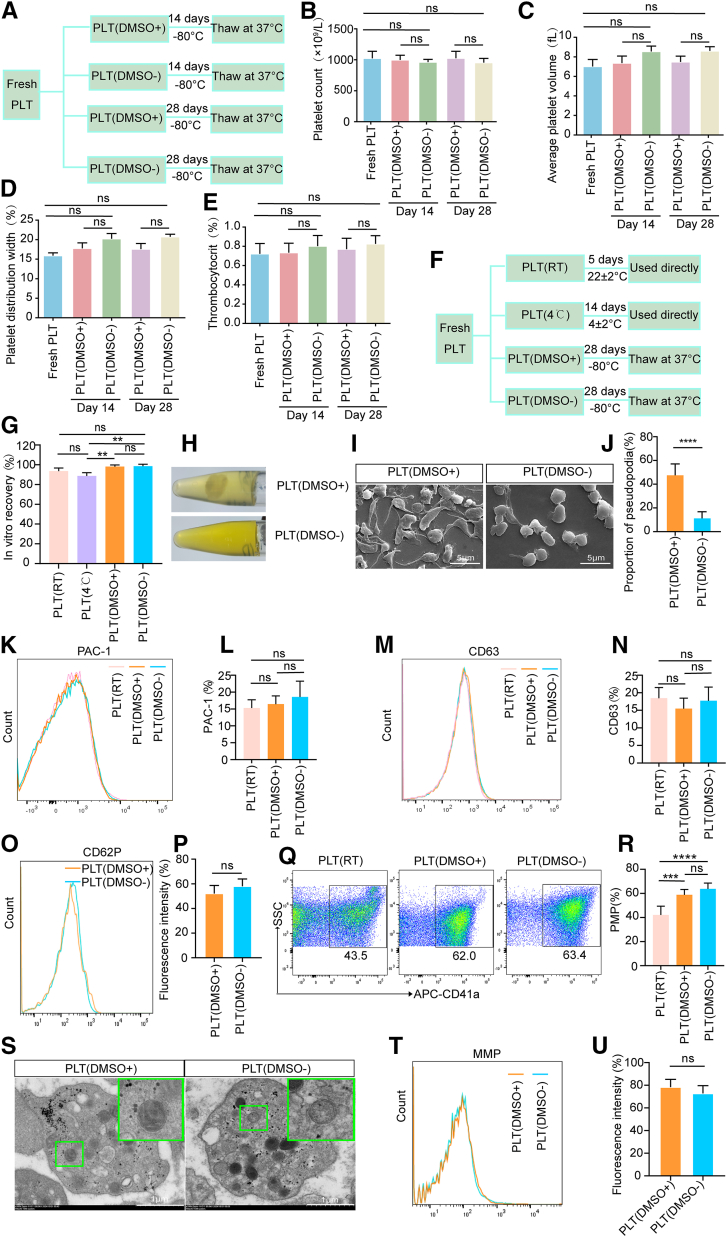
Quality analysis of platelets under different storage conditions. (A) Schematic of the experimental workflow for the PLT(DMSO^+^) and PLT(DMSO^−^) groups: platelets were cryopreserved at −80 °C for 14 or 28 days. (B–E) Analysis of platelet count, mean volume, thrombocytocrit, and distribution width for the groups defined in Figure 1A (*n* = 6; ns, no significance). (F) Schematic of the platelet processing workflow for the 4 groups: the −80 °C cryopreserved groups PLT(DMSO^+^) and PLT(DMSO^−^), and the liquid-stored groups at 4 ± 2 °C (PLT[4 °C]) and 22 ± 2 °C (PLT[RT]). (G) *In vitro* recovery rate of platelets in groups defined in Figure 1F (*n* = 6; ns, no significance; ∗∗*P* < .01). (H) Representative images of platelets from the PLT(DMSO^+^) and PLT(DMSO^−^) groups after cryopreservation at −80 °C and thawing at 37 °C. (I, J) Representative scanning electron microscopy images and quantitative analysis of platelets from the PLT(DMSO^+^) and PLT(DMSO^−^) groups after cryopreservation at −80 °C (*n* = 6; ns, no significance; ∗∗∗∗*P* < .0001). (K–N) Representative flow cytometry histograms and quantitative analysis of PAC-1 and CD63 expression levels in the PLT(DMSO^+^), PLT(DMSO^−^), and PLT(RT) groups (*n* = 6; ns, not significant). (O, P) Representative flow cytometry histograms and quantitative analysis of CD62P expression levels in the PLT(DMSO^+^) and PLT(DMSO^−^) groups (*n* = 6; ns: no significance). (Q, R) Quantitative analysis and representative flow cytometry plots of PMP generation for the PLT(DMSO^+^), PLT(DMSO^−^), and PLT(RT) groups (*n* = 6; ns, no significance; ∗∗∗*P* < .001; ∗∗∗∗*P* < .0001). (S) Representative transmission electron microscopy images showing platelet ultrastructure. (T, U) Representative flow cytometry histograms and quantitative analysis of MMP fluorescence intensity in the PLT(DMSO^+^), PLT(DMSO^−^), and PLT(RT) groups (*n* = 6; ns, no significance). PLT(RT), platelets stored at 22 ± 2 °C for 5 days; PLT(4 °C), platelets stored at 4 ± 2 °C for 14 days; PLT(DMSO^+^), platelets in plasma with 5% DMSO; PLT(DMSO^−^), platelets in plasma without DMSO. DMSO, dimethyl sulfoxide; MMP, mitochondrial membrane potential; PLT, platelet; RT, room temperature.

Following a 2-hour resting period at 22 ± 2 °C, the platelet concentrates were subsequently agitated at 22 ± 2 °C for 5 hours and diluted to a concentration of 9∼10 × 10^8^/mL. The samples were then randomly allocated into 4 experimental groups with distinct storage protocols. Groups 1 and 2 were stored in gas-permeable platelet storage bags (5 mL; Fresenius Kabi) under the following conditions: (1) platelets stored at 22 ± 2 °C for 5 days; (2) platelets stored at 4 ± 2 °C for 14 days. Groups 3 and 4 were designated for cryopreservation studies: (3) the PLT(DMSO^+^) group, where 5% DMSO was added as a cryoprotectant; and (4) the PLT(DMSO^−^) group (plasma alone without DMSO). Samples from these 2 groups were aliquoted into 1.5-mL microcentrifuge tubes (1 mL per tube; Sangon Biotech) and stored at –80 °C for either 14 or 28 days. For subsequent analysis, frozen samples from groups 3 and 4 were rapidly thawed in a 37 °C water bath with gentle agitation for 1 to 2 minutes, whereas samples from groups 1 and 2 were processed immediately upon completion of their respective storage periods. The overall experimental design and storage scheme are summarized in Figure 1A.

The study protocol received approval from the Ethics Committee of Xijing Hospital (approval number: KY20252497-F-1), and all human participants provided written informed consent. The study was conducted in accordance with the Declaration of Helsinki. All experimental procedures for humans were performed according to the requirements of the Ethics Committee of Xijing Hospital.

### 2.2 Analysis of platelet parameters

Platelet parameters, including numbers, average volume, thrombocytocrit, and distribution width, were assessed using an automatic hematology analyzer (XP-100; Sysmex). Measurements of platelet parameters stored at −80 °C were conducted after 14 and 28 days of cryopreservation, in addition to evaluations of fresh apheresis platelets.

### 2.3 Analysis of platelet morphology

Platelet morphology was examined by scanning electron microscopy (SEM; Thermo Scientific Quattro S) and transmission electron microscopy (TEM; HT7800; HITACHI). For SEM, 50 μL of platelet suspension (5 × 10^8^/mL) was mixed with 1 mL of 2.5% glutaraldehyde in 0.1 M phosphate buffer and fixed for 2 hours. The samples were then rinsed 5 times (6 minutes each) with 0.1 M phosphate buffer (pH 7.2), followed by dehydration through a graded acetone series: 30%, 50%, 70%, and 90% acetone for 30 minutes each, and 100% acetone 3 times for 10 minutes each. The samples were subsequently dried using the critical point drying method with CO_2_, sputter-coated with gold, and observed under SEM. For TEM, 1 mL of platelet suspension (5 × 10^8^/mL) was pelleted and fixed with 2.5% glutaraldehyde in 0.1 M cacodylate buffer at 4 °C for 2 hours. All samples were then postfixed in 1% osmium tetroxide, dehydrated through a graded ethanol series, and embedded in epoxy resin. Ultrathin sections were examined by TEM. These samples were processed at the Electron Microscopy Center of the Fourth Military Medical University following previously reported protocols [22]. All experiments were performed in 3 independent biological replicates, with 3 random fields of view captured per group.

### 2.4 Analysis of phosphatidylserine exposure

Platelet phosphatidylserine (PS) exposure was measured using an Annexin V kit (catalog No. 556547; BD Biosciences). Platelets (1 × 10^6^/mL) were suspended in 1× binding buffer (10 mM HEPES [pH 7.4], 140 mM NaCl, 2.5 mM CaCl_2_) [23] after the different storage conditions described earlier. Then, 1 μL Annexin V was added into 19 μL diluted platelets, and the suspension was incubated in dark at 22 °C for 15 minutes for staining. After incubation of the samples, the mixed suspension was washed with 1× binding buffer. Finally, the samples were resuspended in 300 μL of 1× binding buffer and analyzed by flow cytometry (FACSCanto; BD Biosciences), with 5000 cells acquired per sample for data analysis.

### 2.5 Analysis of platelet activation

Platelet activation was analyzed by measuring the expression of CD62P, PAC-1 (GPIIb/IIIa), and CD63 as described in our previous study [22]. Briefly, 1 μL of each respective antihuman antibody (anti-CD62P: catalog No. 5555523; BD Biosciences; anti–PAC-1: catalog No. 62803; biolegend; and anti-CD63: catalog No. 353005; biolegend) was added to 19 μL samples for staining. The samples were then incubated in the dark at 22 °C for 15 minutes, washed with 1× binding buffer postincubation, and resuspended in 300 μL of 1× binding buffer. Finally, all stained samples were analyzed by flow cytometry (FACSCanto), with 5000 cells acquired per sample for data analysis.

### 2.7 Analysis of mitochondrial membrane potential

The mitochondrial membrane potential (MMP) of platelets was measured using the MMP assay kit with TMRE (catalog No. C2001S; Beyotime). First, TMRE fluorescent probe (tetramethylrhodamine ethyl ester) was diluted with MMP detection buffer at a ratio of 1:1000 to a final concentration of 10 μM. Second, the number of platelets was adjusted to 1 × 10^9^/mL with phosphate-buffered saline buffer for preparation. After taking 5 μL of platelet suspension, 100 μL of the MMP detection probe at a concentration of 10 μM was added, thoroughly mixed, and incubated at room temperature in a dark place for 20 minutes. Finally, the reaction was terminated by adding 400 μL of detection buffer, and samples were then analyzed by flow cytometry with fluorescence set to the PE channel (FACSCanto), with 5000 cells acquired per sample for data analysis.

### 2.8 Metabolomic analysis and mass spectrometry

Platelets that had been stored at −80 °C for 28 days as described earlier underwent metabolomics analysis using high-performance liquid chromatography-tandem mass spectrometry (LC-MS) at Aptikon Biological. Platelets that had been stored at −80 °C for 28 days as described earlier underwent proteomic analysis using LC-MS/MS at Aptikon Biological. The detailed experimental analysis methods of metabolomics and proteomics are shown in the Supplementary Material 2.

### 2.9 Thromboelastography analysis

Platelet coagulation function was evaluated using a kaolin-activated thromboelastography (TEG) assay on a TEG analyzer (ATEG-8; Changjiang Biotechnology) in accordance with the manufacturer’s instructions. Briefly, platelets were reconstituted with red blood cells of the identical blood type to achieve a final platelet concentration of 2 × 10^11^/mL. For the TEG measurement, 340 μL of this reconstituted sample was combined with 10 μL of a 4% kaolin activator and incubated at room temperature for 5 minutes to allow for contact activation. Subsequently, 20 μL of 0.2 M calcium chloride was dispensed into a prewarmed TEG assay cup, followed by the immediate addition of 340 μL of the kaolin-activated mixture to initiate recalcification and clot formation. The TEG tracing was continuously monitored at 37 °C until a stable maximum amplitude was attained.

### 2.10 Analysis of platelet-derived microparticles

Platelet-derived microparticles (PMPs) were analyzed by flow cytometry. Platelet-free plasma was prepared from all experimental groups by centrifugation at 2000 × *g* for 20 minutes. A fixed volume of 50 μL of platelet-free plasma from each sample was incubated with an anti-CD41a antibody (catalog No. 561852; BD Biosciences) for 20 minutes in the dark at room temperature. After staining, samples were diluted with 1× binding buffer and analyzed immediately on a FACSCanto flow cytometer. The percentage of CD41a-positive events within the total acquired events was determined for each sample. Results are presented as the relative proportion of PMPs (%) in an equal volume (50 μL) of platelet-free plasma across the different experimental groups.

### 2.11 Analysis of the clot retraction function of platelets *in vitro*

To assess clot retraction, a 500-μL aliquot of platelet suspension from each group was transferred into a prewarmed tube. CaCl_2_ and thrombin were then added to achieve final concentrations of 0.025 M and 0.5 U/mL, respectively, to initiate clotting. The reaction mixture was incubated undisturbed at 37 °C. The formed clot was photographed at specified time points (0, 20, 40, and 60 minutes). The retracted clot area from each image was subsequently quantified using ImageJ software (National Institutes of Health).

### 2.12 Analysis of the adhesion function of platelets *in vitro*

Briefly, washed platelets (1 × 10^7^/mL) were seeded onto 24-well plates coated with type I collagen (100 μg/mL; Yuanye) or fibrinogen (100 μg/mL; Sigma) and incubated for 1 hour at 37 °C. Following phosphate-buffered saline washing, adherent platelets in 3 random fields per well were counted using an inverted microscope (ECLIPSE; Nikon) and normalized to the day 0 control.

### 2.13 Analysis of *in vitro* platelet recovery rate

Following storage under the designated conditions, platelet counts for each of the 4 storage groups were measured using an automatic hematology analyzer (Sysmex XP-100). The *in vitro* platelet recovery rate was calculated as the poststorage platelet count divided by the baseline platelet count (day 0) for each respective sample.

### 2.14 Animal studies and *in vivo* bleeding assay

NOD-SCID (non-obesediabetic/ShiLtJGpt-Prkdc^em26Cd52^/Gpt) mice were purchased from GemPharmatech (Strain No. T001492) and housed in a specific pathogen-free environment. Sex- and age-matched (10-12 weeks) animals were used in this study. Mice were randomly assigned to experimental groups using a random number generator. The operator was blinded to the group allocation throughout the experiment. Data analysis was performed by an independent investigator who was unaware of the group assignments. Five mice were included in each experimental group for all *in vivo* studies. All animal protocols and ethics statements were reviewed and approved by the Animal Resources Center of Fourth Military Medical University (approval number: 20250116). NOD-SCID mice were anesthetized with pentobarbital sodium (0.05 mg/g, intraperitoneal injection) prior to the procedure. To deplete autologous platelets, antimouse CD42b monoclonal antibody (0.5 μL/g; catalog No. R300; Emfret) was administered via the tail vein, followed by infusion of stored human platelets (1 × 10^9^/mL, 18 μL/g). Subsequently, bleeding was induced by amputating a 2-mm segment from the tail tip using a sterile scalpel.

To measure bleeding time, the transected tail was immersed vertically into 1 mL of prewarmed saline (37 °C) in an EP tube. Timing began at the moment of amputation and ceased upon complete hemostasis, defined as the absence of bleeding for 30 consecutive seconds. If bleeding did not stop within 50 minutes, the observation was discontinued, and the bleeding time was recorded as 50 minutes for analysis. Bleeding duration was recorded accordingly. The total volume of the resulting mixture (saline plus shed blood) was then measured, and blood loss volume (in milliliters) was calculated by subtracting the initial 1 mL saline volume from this total. At the end of the experiment, all mice were humanely euthanized by cervical dislocation, performed by trained personnel proficient in this technique in accordance with institutional animal welfare guidelines.

### 2.15 Analysis of *in vivo* platelet survival

For platelet clearance analysis, 5 mice were included in each experimental group. Stored human platelets were injected into NOD-SCID mice via the tail vein (1 × 10^9^/mL, 14 μL/g) using the same randomization and blinding protocols as described earlier. Subsequently, peripheral blood was collected at 8, 30, 60, and 120 minutes postinjection. The samples were labeled with FITC-conjugated anti-human CD41 (catalog No. 362804; BioLegend) and analyzed by flow cytometry (FACSCanto). At the end of the experiment, all mice were humanely euthanized by cervical dislocation.

### 2.16 Immunofluorescence analysis

At 4 hours after injection of stored human platelets, NOD-SCID mice were sacrificed to collect liver and spleen, and paraffin sections were prepared. After that, paraffin sections were subjected to antigen retrieval and stained by antihuman CD41 for immunofluorescence analysis of platelets by using an Olympus FluoViewFV1000 confocal laser scanning microscope. All experiments were performed in 3 independent biological replicates, with 3 random fields per group.

### 2.17 Statistical analysis

Data analysis was conducted with GraphPad Prism 8.0 (GraphPad Software). Where appropriate, a paired *t*-test was used for 2-group comparisons. For comparisons across >2 groups, 1-way analysis of variance (anova) was used, followed by Tukey multiple comparisons test. Results are expressed as mean ± SEM. Differences were considered significant at *P* < .05, and significance levels are denoted as ∗*P* < .05, ∗∗*P* < .01, ∗∗∗*P* < .001, and ∗∗∗∗*P* < .0001. The sample size (*n*) indicated in all figures represents the number of biological replicates. The number of independent experiments for each assay is detailed in the corresponding figure legends. All experiments and analyses were performed under blinded conditions to minimize bias. The experimental design and group allocations for all assays are provided in Supplementary Table 1.

## 3 Results

### 3.1 Platelet characteristics were steady at −80 °C in plasma without cryoprotectant

**Supplementary Figure 1:**
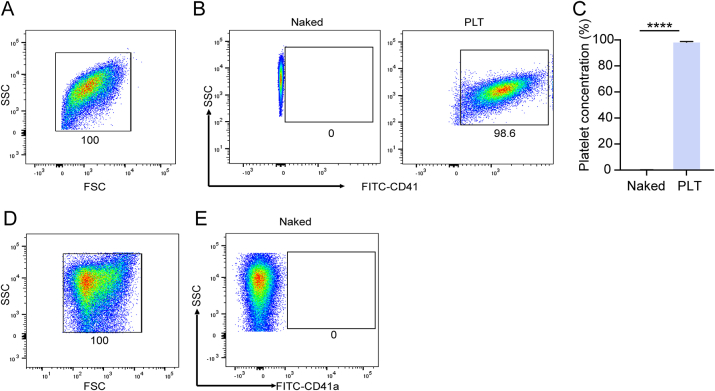


To investigate the impact of cryopreservation on platelet characteristics, we analyzed various parameters using a hematology analyzer. After isolation of highly concentrated platelets, they were cryopreserved at −80 °C with 5% DMSO (PLT[DMSO^+^]) or without DMSO (PLT[DMSO^−^]) for 14 or 28 days (Figure 1A, Supplementary Material 2, Supplementary Figure 1A–C). Our results indicated that platelet count, mean volume, distribution width, and thrombocytocrit remained unchanged after storage at −80 °C compared with fresh platelets, regardless of the presence of DMSO or storage duration (both day 14 and day 28) (Figure 1B–E). Notably, even in the absence of DMSO (PLT[DMSO^−^]), these parameters remained stable at both day 14 and day 28, indicating that basic platelet characteristics are preserved under these conditions.

We next conducted a comprehensive comparison of platelet quality across different storage methods. Two cryopreserved groups stored at −80 °C for 28 days (PLT[DMSO^+^] and PLT[DMSO^−^]) were evaluated alongside 2 conventional liquid-storage groups: PLT(room temperature [RT]) (22 ± 2 °C for 5 days) and PLT(4 °C) (4 ± 2 °C for 14 days), as outlined in Figure 1F. Analysis of *in vitro* platelet recovery rates showed no significant differences among PLT(DMSO^+^), PLT(DMSO−), and PLT(RT), all of which exhibited substantially higher recovery than the PLT(4 °C) group (Figure 1G). Moreover, following rapid thawing at 37 °C, morphologic assessment further revealed an increased number of agglutinated platelets in the PLT(DMSO^+^) group compared with that in the PLT(DMSO^−^) group (Figure 1H). Consistent with this, SEM showed that while platelet morphology remained intact in the PLT(DMSO^−^) group, the PLT(DMSO^+^) group exhibited a greater number of pseudopodia (Figures 1I, J), which may contribute to platelet agglutination. Collectively, these data suggest that platelets stored in plasma without cryoprotectants at −80 °C maintain their basic morphology, while DMSO induces greater agglutination. To determine whether these changes were associated with platelet activation, we measured key activation markers (PAC-1, CD63, and CD62P). Levels of PAC-1 and CD63 were comparable between the cryopreserved groups (PLT[DMSO^+^] and PLT[DMSO^−^]) and the PLT(RT) group (Figure 1K–N). Furthermore, CD62P levels did not differ significantly between the 2 cryopreserved groups (Figure 1O, P). These results indicate that the activation status of platelets in plasma remains unchanged after storage at −80 °C, regardless of DMSO. Finally, we analyzed PMP generation in PLT(DMSO^+^), PLT(DMSO^−^), and PLT(RT), a key parameter of platelet membrane integrity and functional potential. Consistent with previous reports, PMP levels were significantly higher in both the PLT(DMSO^+^) and PLT(DMSO^−^) groups than in the PLT(RT) group. In contrast, PMP generation did not differ significantly between the PLT(DMSO^+^) and PLT(DMSO−) groups (Figure 1Q, R, Supplementary Material 2, Supplementary Figure 1D, E). Therefore, the observed increase in PMPs appears to be primarily a consequence of the cryopreservation and thawing cycle, rather than an effect specific to DMSO.

The abovementioned analyses demonstrated the preservation of basic platelet characteristics and activation status, with PMP generation linked to cryopreservation rather than DMSO. We next evaluated mitochondrial function, a core determinant of platelet vitality and functional competence [[24], [25]]. Using TEM, we observed that platelets in the PLT(DMSO^−^) group maintained relatively intact mitochondrial ultrastructure (Figure 1S). The depolarization of the mitochondrial membrane is recognized as an early indicator of mitochondrial damage. Consequently, we aimed to assess the platelet MMP under various storage conditions. The results indicated no statistically significant differences in MMP levels between the PLT(DMSO^+^) and PLT(DMSO^−^) groups (Figure 1T, U). Collectively, these data suggest that platelets stored in plasma at −80 °C without DMSO-preserved normal mitochondrial structure and MMP levels.

### 3.2 Few impacts on platelet metabolites were observed after storage at −80 °C without cryoprotectants

**Figure 2:**
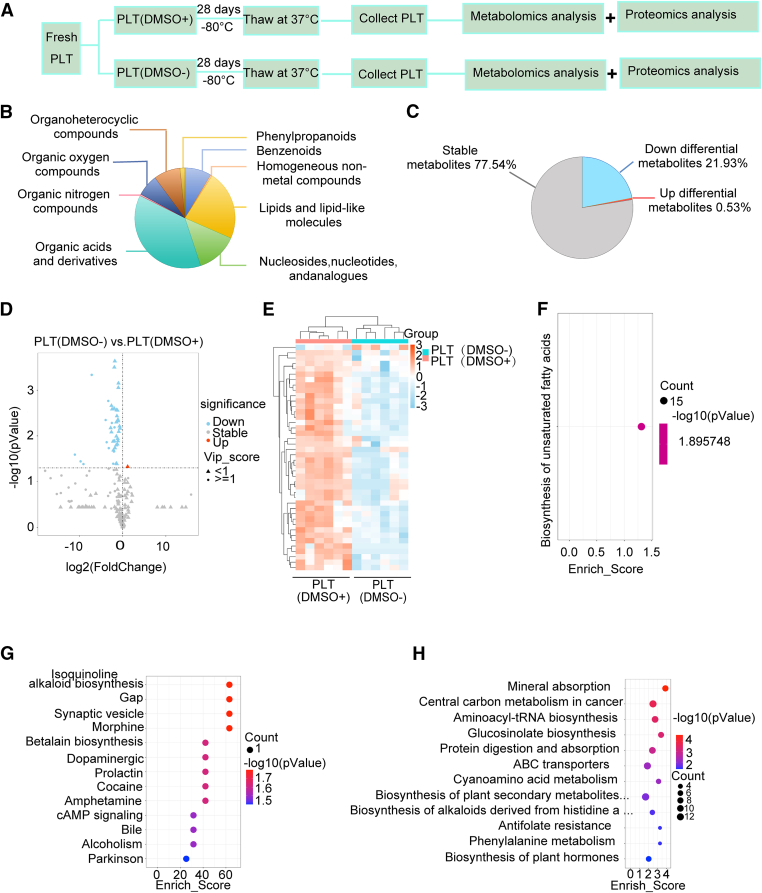
Metabolic profiles of platelets from 6 volunteer donors. (A) Following 28-day storage in plasma, platelets from the PLT(DMSO^+^) and PLT(DMSO^−^) groups were thawed at 37 °C for metabolomic profiling. (B) Classification of metabolites based on metabolic pathways. (C) Proportion of stable, upregulated, and downregulated metabolites in platelets stored in plasma alone compared with those stored with DMSO. (D) Volcano plot of differential metabolites. The Benjamini–Hochberg false discovery rate (FDR) method was used to account for multiple comparisons. Metabolites with a fold change of <0.5 or >2 and an adjusted *P* value (FDR < 0.05) were considered significantly decreased (blue) or increased (red). Changes in other metabolites were not significant (gray). Variable importance in projection (VIP) scores from the OPLS-DA model are shown. Triangles represent VIP < 1, and circles indicate VIP ≥ 1 (based on fold change and 2-tailed unpaired Student’s *t*-test or Mann–Whitney U-test). (E) Heatmap of 42 differential metabolites across individual samples. Red indicates metabolites that were increased, and blue indicates metabolites that were decreased in the PLT(DMSO^+^) group compared with the PLT(DMSO^−^) group. (F–H) All metabolites were annotated based on the KEGG database, and the differentially abundant metabolites were subjected to enrichment analysis. The hypergeometric distribution was used to test for significant enrichment. The *x*-axis represents the enrichment score, and the *y*-axis represents different pathways. The color of the circles indicates enrichment significance, and the dot size reflects the number of metabolites (larger dots indicate more metabolites). DMSO, dimethyl sulfoxide; OPLS-DA, orthogonal partial least squares-discriminant analysis; PLT, platelet.

To better understand the profile of platelets stored at −80 °C, metabolomics analyses were conducted in the current study (Figure 2A). Following MS/MS identification and data filtering, 187 metabolites were reproducibly detected across all batches. These identified metabolites were categorized into 9 functional groups based on their metabolic pathways (Figure 2B). The pie chart showed that in the PLT(DMSO^−^) group, 77.54% of metabolites remained stable, while 21.93% decreased, and only 0.53% increased (Figure 2C). The volcano plot visualized the significantly increased or decreased metabolites between PLT(DMSO^+^) and PLT(DMSO^−^) groups through univariate analysis, identifying 42 metabolites that significantly contributed to the variation (Figure 2D). In comparison with the PLT(DMSO^+^) group, the PLT(DMSO^−^) group exhibited 145 metabolites with no significant differences. Only dopamine increased, while 41 metabolites decreased in the PLT(DMSO^−^) group. Upon examining the variable importance in projection, 12 metabolites—including xanthine, uridine, arginine, indole acetic acid, proline, taurine, 3-pyridine acetic acid, lactic acid, myristate, linolenic acid, glutathione, and asymmetric dimethylarginine—were found to be significant (variable importance in projection > 1). All 42 diverse metabolites were used for hierarchical clustering in the heatmap (Figure 2E, Supplementary Material 2, Supplementary Table. 2). All 145 unchanged metabolites could be categorized within the biosynthesis of unsaturated fatty acids pathway (Figure 2F), which plays a crucial role in platelet preservation. The significantly decreased metabolites were associated with the pathways of protein digestion and absorption, glucosinolate biosynthesis, aminoacyl-tRNA biosynthesis, central carbon metabolism in cancer, and mineral absorption (Figure 2G, H). Collectively, these data suggest that platelets stored at −80 °C exhibit similar metabolic characteristics, despite some differences in biosynthesis and protein digestion.

### 3.3 Platelet proteins remained stable after storage at −80 °C in plasma without cryoprotectants

**Figure 3:**
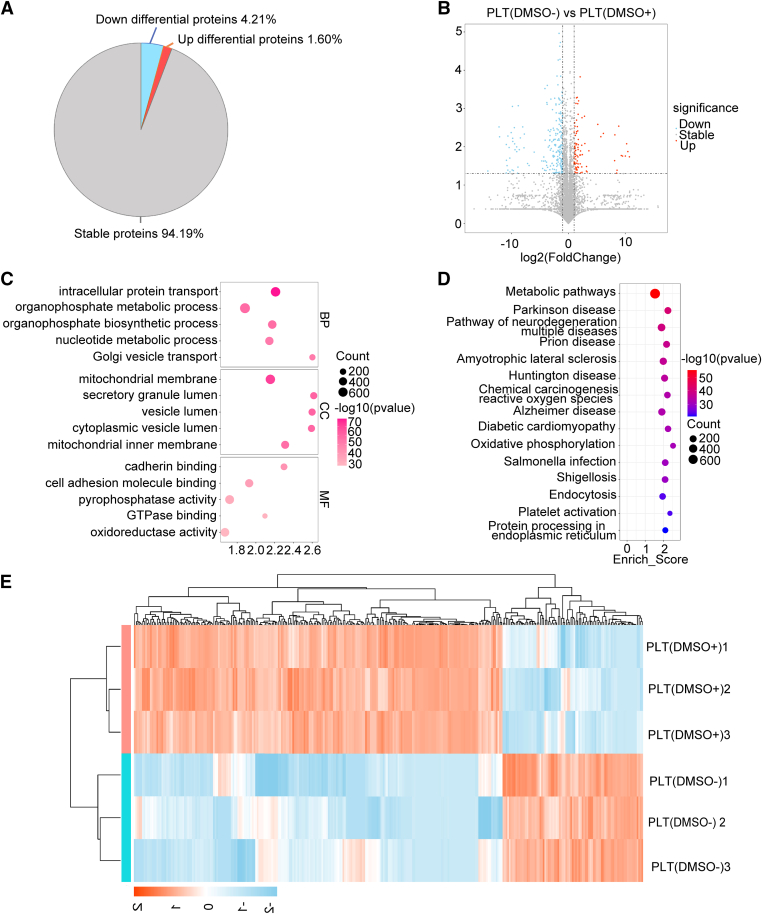
Proteomic analysis of platelets after storage at −80 °C. (A) Distribution of platelet proteins in the PLT(DMSO^+^) and PLT(DMSO^−^) groups. (B) Volcano plot of differentially expressed proteins from stored platelets. Proteins with a fold change of <0.5 or >2 and an adjusted *P* value of <.05 were considered significantly decreased (blue) or increased (red). Changes in other proteins were not significant (gray). (C) Bubble chart showing Gene Ontology enrichment analysis of unchanged proteins from stored platelets. (D) Bubble chart showing Kyoto Encyclopedia of Genes and Genomes enrichment analysis of unchanged proteins from stored platelets. (E) Heatmap of differentially expressed proteins from stored platelets. DMSO, dimethyl sulfoxide; PLT, platelet.

**Supplementary Figure 2:**
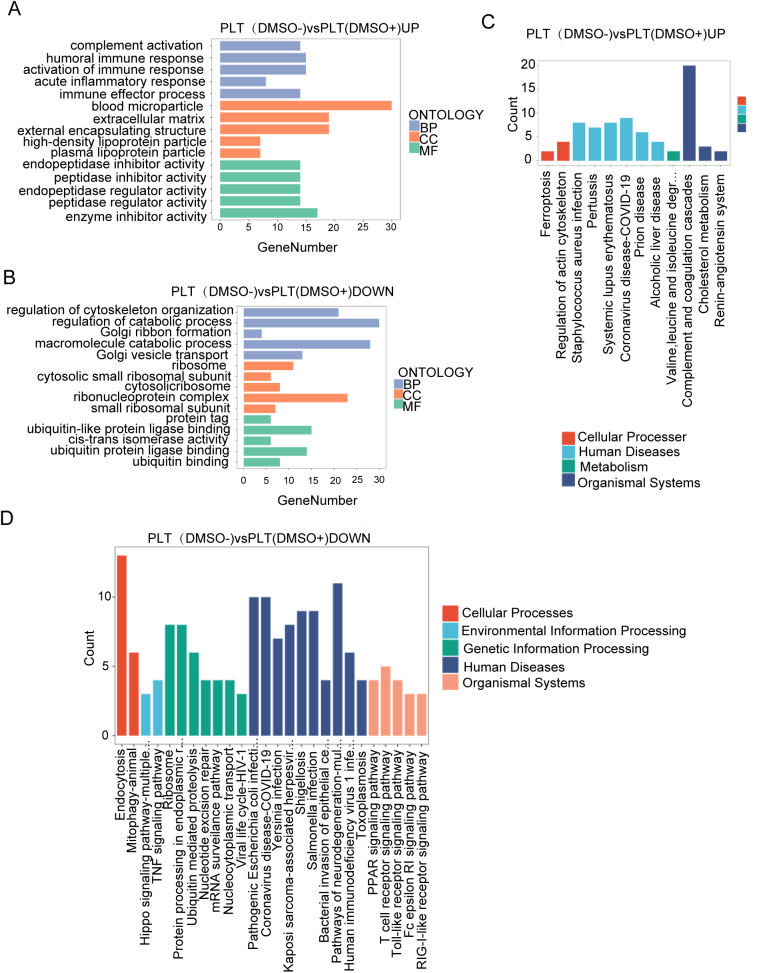


To better understand the profile of platelets stored at −80 °C, a proteomic analysis was subsequently conducted (Figure 2A). Among the 5422 proteins identified in the stored platelets, 5107 proteins (94.19%) exhibited no significant differences, while 87 proteins (1.6%) were upregulated and 228 proteins (4.21%) were downregulated (Figure 3A). The volcano plot illustrated the number of upregulated and downregulated proteins (Figure 3B). Gene Ontology annotation indicated that the stable proteins were primarily associated with cell adhesion molecule binding, cadherin activity, oxidoreductase activity, and pyrophosphatase activity. Additionally, these proteins were involved in Golgi vesicle transport and intracellular protein transport (Figure 3C). Kyoto Encyclopedia of Genes and Genomes (KEGG) annotation suggested that the unchanged proteins from stored platelets were predominantly related to metabolic pathways, platelet activation, oxidative stress, and phosphorylation (Figure 3D). We further investigated the functions of the differential proteins from stored platelets. As shown in Figure 3E, all 315 changed proteins were used for hierarchical clustering in the heatmap. Gene Ontology enrichment analysis demonstrated that the altered proteins were primarily involved in complement activation, blood microparticle formation, endopeptidase inhibitor activity, regulation of cytoskeleton organization, and protein tagging (Supplementary Material 2, Supplementary Figure 2A, B). KEGG annotation revealed that the altered proteins were mainly associated with infection, endocytosis, mitophagy, and ribosome function (Supplementary Material 2, Supplementary Figure 2C, D). Collectively, these data suggest that platelets stored in plasma without DMSO did not exhibit changes in proteins primarily responsible for activation, oxidative stress, or metabolism.

### 3.4 Platelet cryopreservation without cryoprotectants maintained coagulation function both *in vitro* and *in vivo*

**Figure 4:**
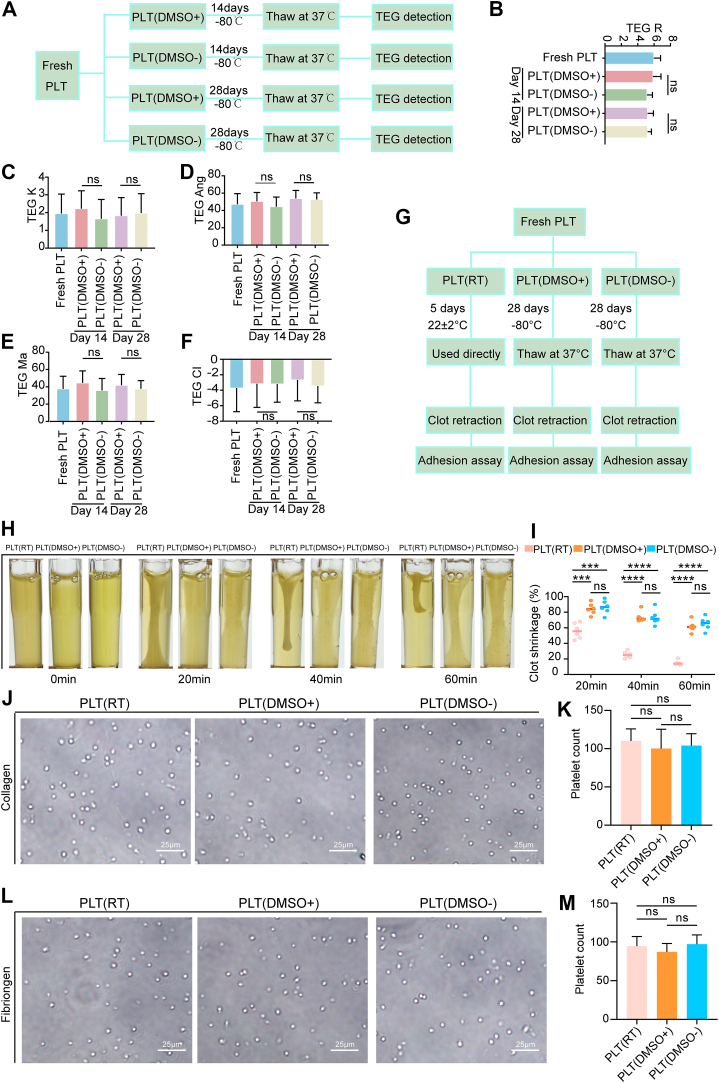
*In vitro* functional assays of platelets from different groups. (A) Schematic of the experimental workflow for the preparation and thromboelastography (TEG) analysis of the PLT(DMSO^+^) and PLT(DMSO^−^) groups cryopreserved at −80 °C for 14 or 28 days. (B–F) TEG parameter analysis of platelets from the experimental groups as defined in Figure 4A (*n* = 6; ns, no significance). (G) Schematic of the platelet processing workflow for the 3 groups (PLT[DMSO^+^], PLT[DMSO^−^], and PLT[RT]) for subsequent clot retraction and *in vitro* adhesion assays. (H, I) Representative images and quantitative analysis of *in vitro* clot retraction for platelets from the PLT(DMSO^+^), PLT(DMSO^−^), and PLT(RT) groups (*n* = 6; ns, no significance; ∗∗∗*P* < .001; ∗∗∗∗*P* < .0001). (J, K) Representative images and quantitative analysis of platelet adhesion to collagen for the PLT(DMSO^+^), PLT(DMSO^−^), and PLT(RT) groups (*n* = 6; ns, no significance). (L, M) Representative images and quantitative analysis of platelet adhesion to fibrinogen for the PLT(DMSO^+^), PLT(DMSO^−^), and PLT(RT) groups (*n* = 6; ns, no significance). DMSO, dimethyl sulfoxide; PLT, platelet; RT, room temperature.

The metabolomic and proteomic analyses indicated that the core molecular profiles of platelets stored under the PLT(DMSO^−^) conditions remained largely unchanged. We therefore evaluated their functional competence *in vitro* and *in vivo*. Thrombus formation was assessed using a simulation model with TEG (Figure 4A). Key TEG parameters (reaction time, clotting time, blood clot formation rate (Angle α), maximum amplitude and coagulation index) showed no significant differences between the PLT(DMSO^−^) and PLT(DMSO^+^) groups (Figure 4B–F), indicating that coagulation function was preserved regardless of DMSO after 28 days at −80 °C. In addition to the overall coagulation profile, we further investigated key platelet-specific functions essential for effective clot formation and stability. Two platelet groups cryopreserved at −80 °C for 28 days (PLT[DMSO^+^] and PLT[DMSO^−^]) were compared with a conventional liquid-storage PLT(RT) group, as outlined in Figure 4G. Our experiments demonstrated no statistically significant difference in platelet adhesion or clot retraction between the PLT(DMSO^+^) and PLT(DMSO^−^) groups (Figure 4H–M). However, while adhesion in both cryopreserved groups was comparable with that in the PLT(RT) group, they exhibited a substantial deficit in clot retraction capacity (Figure 4H, I). Together, these findings establish that cryopreservation at −80 °C without DMSO (PLT[DMSO^−^]) maintains platelet coagulation and adhesion functions equivalent to DMSO-preserved platelets.

**Figure 5:**
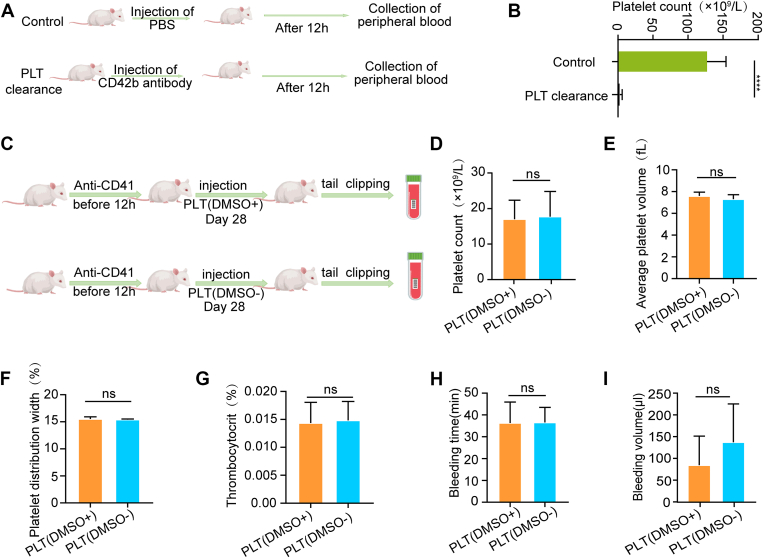
*In vivo* functional assays of platelets from different groups. (A) Anti-CD42b monoclonal antibody was administered to NOD-SCID mice 12 hours before transfusion. (B) Platelet counts after antibody injection (*n* = 5; ∗∗∗∗*P* < .0001). (C) Schematic diagram of the experimental design for functional assessment in NOD-SCID mice following platelet depletion with anti-CD42b antibody. (D–G) Coagulation parameters after transfusion of recovered platelets (*n* = 5; ns, no significance). (H) Bleeding time after partial tail amputation (*n* = 5; ns, no significance). (I) Blood loss volume after partial tail amputation (*n* = 5; ns, no significance; ∗∗*P* < .01). DMSO, dimethyl sulfoxide; PLT, platelet.

To verify the validity of these observations, we established a NOD-SCID mouse model with platelet depletion using CD42b antibody (Figure 5A, B). Following platelet transfusion in these animals, platelet counts, average volume, platelet distribution width, and thrombocytocrit were comparable between the 2 groups (Figure 5C–G). Furthermore, there were no significant differences in bleeding time (Figure 5H) and blood loss volume (Figure 5I) after platelet transfusion in the mice. Collectively, these results demonstrate that platelets stored solely in plasma, without DMSO, maintain their coagulation function both *in vitro* and *in vivo*.

### 3.5 The survival of platelets after cryopreservation was similar with or without cryoprotectants both *in vitro* and *in vivo*

**Figure 6:**
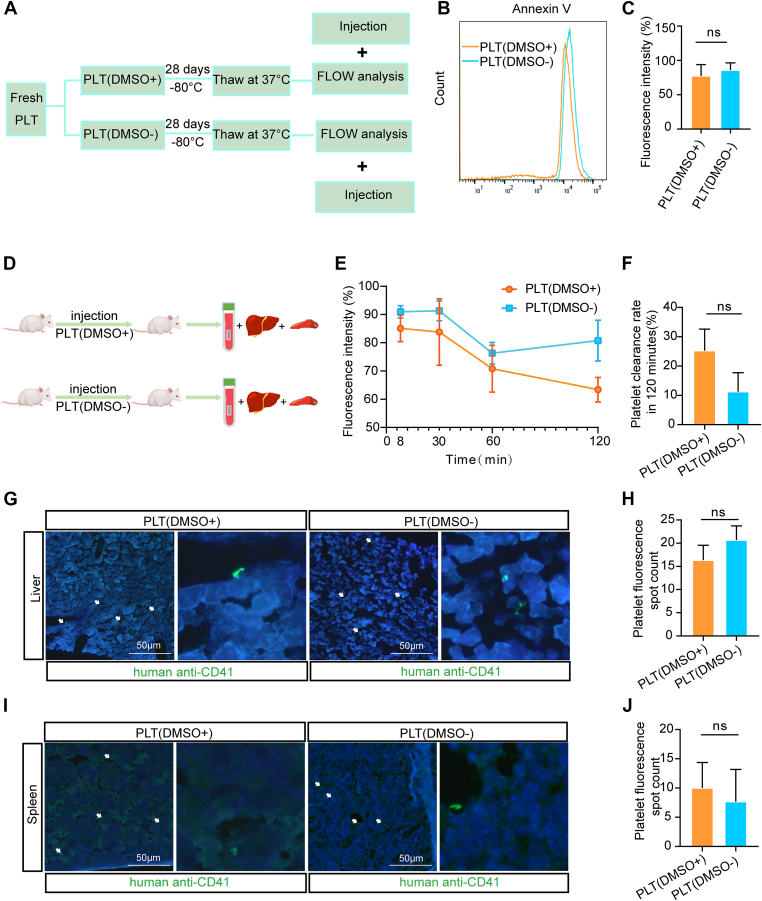
The clearance test of stored platelets *in vitro* and *in vivo*. (A) Schematic diagram of the platelet clearance assay. (B, C) CD62P expression was detected by flow cytometry and quantitatively analyzed (*n* = 5; ns, no significance). (D) Schematic diagram of the platelet evacuation assay after transfusion in mice. (E) Viable platelet ratios were analyzed by flow cytometry at different time points after transfusion. (F) Platelet evacuation rate after transfusion. (G, I) Liver and spleen tissue sections were immunostained with an anti-CD42b antibody to detect platelets in the recipient mice after transfusion. (H, J) Quantification of platelet-positive areas in (G) and (I) (*n* = 5; ns, no significance). DMSO, dimethyl sulfoxide; PLT, platelet.

The lifespan of platelets *in vivo* is no >10 days [26]. When damaged, they undergo apoptosis-like changes, characterized by the externalization of PS on the cell surface [27]. We therefore used flow cytometry to detect PS exposure via Annexin V binding in the 2 cryopreserved groups (Figure 6A). The results showed no significant difference in PS externalization between the PLT(DMSO^−^) and PLT(DMSO^+^) groups (Figure 6B, C), suggesting that cryopreservation with or without DMSO does not differentially affect this marker of platelet viability.

The viability and turnover of platelets *in vivo* are crucial for platelets. To evaluate platelet survival and clearance in our system, we analyzed peripheral blood, as well as liver and spleen tissues, from transfused animals (Figure 6D). Analysis of peripheral blood revealed that although platelet viability and counts declined over time, no statistically significant differences were observed between the PLT(DMSO^−^) and PLT(DMSO^+^) groups (Figure 6E, F). Moreover, the number of platelets localized to the liver and spleen was comparable between the 2 groups (Figure 6G–J). Together, these findings indicate that the PLT(DMSO^−^) storage strategy does not alter platelet turnover or clearance *in vivo* compared with the PLT(DMSO^+^) strategy.

### 3.6 The lifespan and function of platelets stored in plasma at 22 ± 2 °C for 5 days followed by cryopreservation were similar to those of platelets directly cryopreserved in plasma

**Figure 7:**
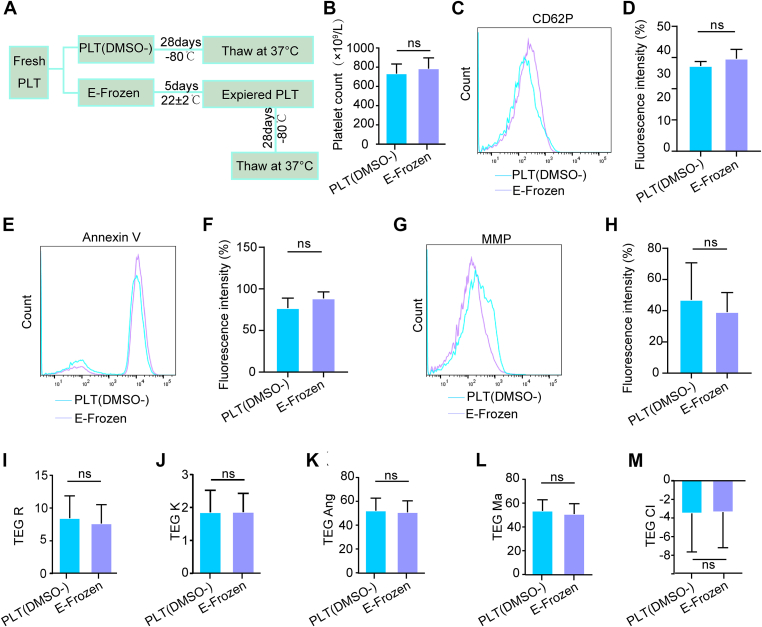
The characters of apheresis platelets after 5 days at 22 ± 2 °C and followed cryopreserved storage in sole plasma at −80 °C. (A) Schematic diagram of the experimental design. (B) Platelet counts under the indicated storage conditions (*n* = 6; ns, no significance). (C, D) CD62P expression was detected by flow cytometry and quantitatively analyzed (*n* = 6; ns, no significance). (E, F) Phosphatidylserine (PS) exposure was measured and quantitatively analyzed (*n* = 6; ns, no significance). (G, H) Mitochondrial membrane potential (MMP) was detected and statistically analyzed (*n* = 6; ns, no significance). (I–M) TEG parameters were analyzed (*n* = 6; ns, no significance). DMSO, dimethyl sulfoxide; PLT, platelet; TEG, thromboelastography.

Platelets stored at 22 ± 2 °C with agitation typically maintain a shelf life of 5 to 7 days [8,[28], [29], [30]]. Due to this limited storage duration, platelets stored without DMSO were previously discarded after 5 days of storage. Given our finding that platelets can be effectively cryopreserved in plasma alone (PLT[DMSO^−^]), we explored the feasibility of applying this method to platelets after they had been stored at 22 °C for 5 days. Following the procedure outlined in Figure 7A, we observed that the recovered platelet count was similar between the PLT(DMSO^−^) group and the E-frozen group (fresh platelets stored at 22 ± 2 °C for 5 days and then at −80 °C for 28 days) (Figure 7B). In addition, the expression of CD62P in platelets in 2 groups did not differ significantly between the 2 groups (Figure 7C, D). Furthermore, PS translocation and MMP were comparable between the 2 groups (Figure 7E–H). Importantly, TEG parameters did not exhibit significant changes in either group (Figure 7I–M). Collectively, these data demonstrate that the functionality of platelets can be partially preserved after 5 days at 22 ± 2 °C followed by cryopreservation.

## 4 Discussion

The preservation of platelets is critical to transfusion medicine. Various methods have been developed to maintain their functionality and viability. In this study, we used plasma alone to preserve platelets and compared various parameters between platelets stored in plasma with and without DMSO. We found that critical aspects of platelets preserved in plasma alone, such as cell count, morphology, metabolic profiles, proteomic characteristics, coagulation function, and survival, were comparable with those of platelets in plasma with DMSO, with minimal alterations in protein composition and metabolic stability. These findings emphasize the potential of this storage method to enhance platelet availability in transfusion medicine, supporting further investigations to optimize conditions and validate clinical applications. Currently, platelets are mainly stored at 22 ± 2 °C for 5 to 7 days [31]. The short shelf-life limitation restricts both the collection and utilization of platelets. On one hand, blood banks are unable to collect platelets in large quantities; if they remain unused within this period, they must be discarded, resulting in a significant waste of valuable blood resources. On the other hand, the clinical application of platelets is constrained; the inability to store them in substantial amounts leads to a chronic shortage of clinical supply [32]. Our findings in this study suggest that platelets stored at 22 ± 2 °C for 5 days can also be cryopreserved at −80 °C for at least 28 days. By extending the storage period through cryopreservation, this approach not only facilitates the collection and preservation of larger quantities of platelets but also ensures a more reliable supply for clinical platelet transfusion, thereby offering more options for precision platelet transfusion. Thus, our method can serve as a valuable supplement to routine clinical platelet preservation.

The survival analysis revealed that platelet cryopreservation at −80 °C does not affect platelet recovery rate in plasma with or without DMSO. However, we should point out that platelets cryopreserved with plasma solely at −80 °C survived *in vivo* for only several hours. In fact, reports on platelets stored at 4 ± 2 °C revealed that they could still be beneficial in trauma, emergency care, or surgery if other platelets were not available in a timely manner [[33], [34]]. Although these findings in the current study underscore the efficacy of plasma storage in preserving platelet functionality and viability, the clinical conditions under which platelets recovered from plasma can be used should be investigated in the future.

Metabolism has gained increasing attention worldwide in the context of platelet storage lesion [[6], [35], [36]]. In the present study, metabolomic analysis provides novel insights into the metabolic pathways altered under different storage conditions. The observation that 77.54% of detectable metabolites remained stable in platelets stored without DMSO suggests that cold storage may maintain metabolic homeostasis to a certain degree, thereby supporting sustained platelet functionality. The elevation in dopamine levels, together with the marked reduction in other metabolites, indicates a shift in metabolic flux that may be critical for platelet activation and responsiveness after transfusion. These results are consistent with previous reports [[15], [37], [38]], suggesting that specific metabolic pathways are preferentially maintained during cold storage, which may alleviate the deleterious effects of platelet storage lesions. Further studies will be required to better characterize these metabolic alterations and their functional consequences on platelet activity, which may help optimize platelet storage strategies based on metabolic signatures.

Although proteins in platelets are less abundant than those in plasma, function-executive proteins are critical for platelet roles in thrombosis and hemostasis [[39], [40], [41]]. The proteomic analysis conducted in this study reinforces the notion that the fundamental protein composition vital for platelet function remains largely intact during cold storage without DMSO. The preservation of 94.19% of proteins indicates that the core functionality of platelets, including their ability to respond to activation signals, is maintained under these conditions. The observed downregulation in specific proteins may provide insights into adaptive responses to storage stress, warranting further investigations into their functional implications. These results emphasize the importance of proteomic stability in posttransfusion recovery and hemostatic function, suggesting that storage methods should be developed to enhance not only the viability but also the functional capacity of stored platelets. Overall, this study provides a comprehensive framework for understanding the effects of storage conditions on platelet biology, which could inform future efforts to improve platelet preservation techniques in clinical settings.

The limitations of this study warrant careful consideration. First, although the NOD/SCID mouse model allows for the tracking of human platelet survival, the inherent interspecies physiological incompatibilities mean that the observed survival kinetics cannot be directly extrapolated to accurately predict human platelet lifespan *in vivo*. Second, while our study assessed key platelet functional endpoints, it did not delve into the underlying intracellular signaling pathways (eg, specific kinase activation and calcium flux), which regulate the observed phenotypic responses to cryopreservation. Third, our evaluation was confined to the efficacy observed after a 28-day preservation period, without an assessment of long-term effects.

In conclusion, this investigation provides evidence that apheresis platelets can be stored in plasma without cryoprotectants at −80 °C for 28 days while largely maintaining functionality. These findings suggest that storing platelets in plasma alone at −80 °C for 28 days offers a potential strategy to extend shelf life beyond the conventional 5-day period, which may help alleviate blood supply shortages.

## Figures and Tables

**Supplementary Figure 3:**
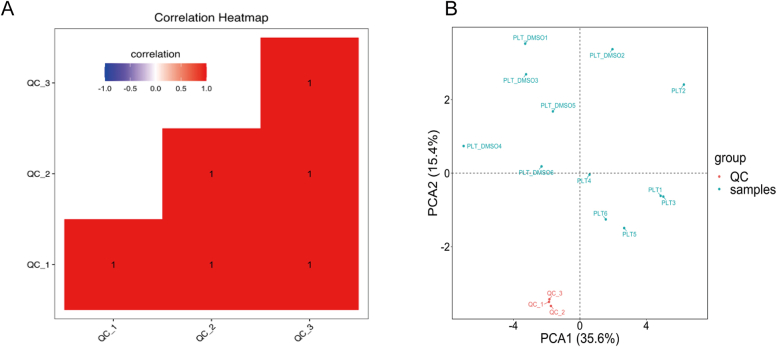


**Supplementary Figure 4:**