# Supplementary material for: The viability and coagulation function of storing platelets can be maintained in plasma without cryoprotectants at −80 °C
Source: Res Pract Thromb Haemost. 2026 May 8;10(4):106635. doi: 10.1016/j.rpth.2026.106635 (PMC13241721; doi:10.1016/j.rpth.2026.106635)
Supplement: Supplementary Material 1 [file mmc1.docx]

## Q450 targeted metabolic quantification method

**1.** Method

**1.1 Extraction of metabolites (fluids)**

(1) Samples are stored at -80°C and thawed at 4°C before use;

(2) Take 50 µL of **fluids** sample (Serum,Urine,et,al) and place it in a 1.5mL-EP tube. Add 200µL of pre-cooled internal standard extraction solution (volume ratio, 4:1).

(3) Vortex for 30 seconds, then ice bath and sonicate for 5 minutes.

(4) Place in a -20°C refrigerator for 1 hour to precipitate proteins and extract metabolites.

(5) Vortex for 30 seconds, then 4 °C centrifuge at 14,000 rpm for 10 minutes.

(6) Take 200µL of the supernatant, and dry it using nitrogen gas or freeze-drying.

(7) Dissolve with 100µL of methanol/water (1:1, V/V).

(8) Vortex for 30 seconds, then ice bath and sonicate for 5 minutes.

(9) 4°C centrifuge at 14,000 rpm for 10 minutes . Place in a sample vial for MS detection.

1.2 Mass spectrometry Detection

RP LC-MS Reference Conditions

| LC Conditions | Parameters |
| --- | --- |
| Injection needle  wash solution | 50%ACN in water |
| Solvent A | water with 0.1% FA |
| Solvent B | acetonitrile with 0.1% FA |
| Column | ACQUITY UPLC HSS T3 1.8 µm 2.1*100 mm column(ABSCIX demo) |
| TCC temperature | 40°C |
| Flow Rate | 0.4mL/min |
| Injector temp. | 8°C |
| Gradient | 0-1 min(5%B)，1-5 min(5-30%B)，5-9 min(30-50%B)，9-12 min(50-75%B)，12-15 min(75-95%B)，15-16 min(95-100%B)16-18 min(100%B)，18-18.1 min(100-5%)，18.1-20 min(5%B)。 |
| MPX Settings | Mass spectrometry acquisition time：0-20min |
| Ion source parameters | Source temperature：550°C.  lon spray voltage：5000V(positve mode), -4500V(negtive mode)。  GS1，GS2：55psi，55psi |
| Scan Mode | Scheduled MRM mode |

(1)l samples are thawed at 4°C and ready for MS detection.

(2)Collect sample data in the following order：

| NO. | Sample  description |
| --- | --- |
| 1 | BlankSample1 (BS1) |
| 2 | Standard 0 (3 needles) |
| 3 | Standard 1-10 （Load samples in the order of the following dilution ratios **1,2,10**,20,40,80,160,320,640,**1280(200uM)** |
| 4 | QC，3 needles |
| 5 | Sample1-10 |
| 6 | BlankSample2 (BS2) |
| 7 | QC |
| 8 | Sample11-20 |
| 9 | ...... |

**Label-free Quantitative Proteomics method**

**1** Methods

**1.1 Protein extraction and trypsin digestion**

**1.1.1 Samples of cell and tissue**

**Protein extraction :**

Lysis buffer [0.1 M Tris-HCl (pH 8.0), 0.1 M DTT (Sigma, 43815), 1 mM PMSF (Amresco, M145)] was added to the extracted tissues, and subsequently sonicated for 1 min (3s on and 3s off, amplitude 25%) on ice. The supernatants were collected, and the extracted tissues were then lysed with 4% sodium dodecyl sulfate (SDS) and kept for 2h at 99°C with shaking at 1,500 rpm. The solution was collected by centrifugation at 12,000×g for 5 min. A 4-fold volume of acetone was added to the supernatant and kept in -20 °C for a minimum of 4 h. Subsequently, the acetone-precipitated proteins were washed three times with cooled acetone and then pumped out using the AQ model Vacuum concentrator (Eppendorf, Germany). The proteins were resuspended in 200 μL 8 M urea (pH 8.0). Protein concentrations were determined by the Bradford protein assay.

**SDS-PAGE :**

20 µg protein for each sample were mixed with 2X loading buffer respectively and boiled for 5 min. The proteins were separated on SDS-PAGE gel (constant current 120 V, 60 min). Protein bands were visualized by Coomassie Blue R-250 staining.

**Filter-aided sample preparation (FASP Digestion)** **procedure**

200 ug protein for each sample were reduced with 10 mM dithiothreitol at 56 °C for 30 min and alkylated with 10 mM iodoacetamide at room temperature in the dark for 30 min. 200μg sample lysis buffer with 200 μl 8M urea bicarbonate in the filter unit, vortex and spin at 14000 rcf for 20 minutes, The filters were washed with 200 μL UA buffer three times and then 200 μl 50mM NH4HCO3 buffer three times. Finally, the protein suspensions were digested with 4 μg trypsin (Promega) in 100 μl 50mM NH4HCO3 buffer overnight at 37 °C, and the resulting peptides were collected as a filtrate. The samples were digested with trypsin using a filter-aided sample preparation method. Tryptic peptides were vacuum-dried (Concentrator Plus, Eppendorf).

1.2 Nano-LC–MS/MS analysis

For the proteome profiling samples, peptides were analyzed on a Orbitrap Fusion Lumos Hybrid Quadrupole-Orbitrap Mass Spectrometer (Thermo Fisher Scientific) coupled with a high-performance liquid chromatography system (EASY nLC 1200, Thermo Fisher Scientific). Dried peptide samples re-dissolved in Solvent A (0.1 % formic acid in water) were loaded onto a 2-cm self-packed trap column (100 μm inner diameter, 3 μm ReproSil-Pur C18-AQ beads, Dr Maisch GmbH) using Solvent A and separated on a 150 μm-inner-diameter column with a length of 15 cm (1.9 μm ReproSil-Pur C18-AQ beads, Dr. Maisch GmbH) over a 120-min gradient (Solvent A: 0.1% formic acid in water; Solvent B: 0.1% formic acid in 80% ACN) at a constant flow rate of 600 nL/min (0–120 min, 0 min, 4% B; 0-8 min, 4–15% B; 8–100 min, 15–35% B; 100–110 min, 30–50% B; 110–111 min, 50–100% B; 111–120 min, 100% B). Eluted peptides were ionized at 2 kV and introduced into the mass spectrometer. Mass spectrometry was performed in data-dependent acquisition mode. For the MS1 Spectra full scan, ions with m/z ranging from 300 to 1,400 were acquired by an Orbitrap mass analyzer at a high resolution of 120,000. The automatic gain control (AGC) target value was set to 3E+06. The maximal ion injection time was 80 ms. MS2 spectral acquisition was performed in the ion trap in a rapid speed mode with 1.5s cycletime. Precursor ions were selected and fragmented with higher energy collision dissociation (HCD) with a normalized collision energy of 30 %. Fragment ions were analyzed by an ion trap mass analyzer with an AGC target at 5E+04. The maximal ion injection time of MS2 was 20 ms. Peptides that triggered MS/MS scans were dynamically excluded from further MS/MS scans for 40 s.

1.3 Data analysis

The original data of mass spectrometry analysis were RAW files, and Proteome Discoverer 2.3 was used for qualitative and quantitative analysis.

| **Item** | **Value** |
| --- | --- |
| **Enzyme** | Trypsin |
| **Database** | See the project report |
| **Fixed modifications** | Carbamidomethyl （C） |
| **Variable modifications** | Oxidation （M）， Acetyl （Protein N-term） |
| **Missed Cleavage** | 2 |
| **Peptide Mass Tolerance** | 20ppm |
| **Fragment Mass Tolerance** | 0.05Da |
