# Supplementary material for: The viability and coagulation function of storing platelets can be maintained in plasma without cryoprotectants at −80 °C
Source: Res Pract Thromb Haemost. 2026 May 8;10(4):106635. doi: 10.1016/j.rpth.2026.106635 (PMC13241721; doi:10.1016/j.rpth.2026.106635)
Supplement: Supplementary Material 2 [file mmc2.docx]

**S-Figure 1. Flow cytometric gating strategy for platelets.**

(A-B) **Human platelets are identified by a specific gating strategy and exhibit high CD41 expression.** (C) Statistical analysis of human platelet concentration (N=3, ****p＜0.0001). (D-E) The gating strategy to identify human PMPs was defined based on size and positivity for platelet-specific markers (CD41a).

**S-Figure 2. Analysis of differential proteins as bar charts.**

(A) Bar chart showing GO enrichment of up-regulated proteins from stored platelets.`(B) Bar chart showing GO enrichment of down-regulated proteins from stored platelets. (C) Bar chart showing KEGG enrichment of up-regulated proteins from stored platelets. (D) Bar chart showing KEGG enrichment of down-regulated proteins from stored platelets.

**S-Figure 3. The quality control of metabolomics.**

(A) QC sample correlation. A Pearson correlation analysis was conducted using QC samples, as shown in the figure below. In this experiment, the correlation coefficients of the QC samples were mostly above 0.9, indicating good experimental repeatability and stable instrument conditions throughout the entire experimental process. (B) QC sample PCA plot. Using unsupervised Principal Component Analysis (PCA), we model the experimental samples and QC samples, and then present the score plots. This intra-group PCA analysis eliminates the interference from different groups, allowing us to more clearly observe the variations within the groups and identify possible outliers. Theoretically, in the data acquisition process without signal drift, the signal strength of the QC samples remains unchanged. Therefore, in the PCA plot, the QC samples should cluster together.

**S-Figure 4. The quality control of proteomics.**

(A) Histogram of peptide count distribution. The horizontal axis represents the number of peptide segments matching the same protein; the main vertical axis (Number of Protein) is the corresponding bar chart for the number of corresponding proteins. The secondary vertical axis corresponds to the cumulative curve in the figure, indicating the cumulative percentage of proteins whose peptide segments are no more than the corresponding value. (B) Histogram of peptide length distribution. The abscissa represents the length of the peptide sequence; the ordinate represents the number of peptide segments within that length range. The length of peptides detected by mass spectrometry is generally distributed within the range of 6 to 25 amino acid sequences. If the amino acid sequence is too short, the number of fragment ions will be insufficient, which will affect the peptide score. If the peptide ion is too long, there will be a significant difference between the actual working resolution of the mass spectrometer and the set parameters, which will affect the credibility of the results.

**Supplemental table**

S-Table 1. Detailed experimental design for all assays

| **Experiment type** | **Total donor pool** | **Donors used (N)** | **Biological replicates** | **Technical replicates** | **Paired design** |
| --- | --- | --- | --- | --- | --- |
| In vitro experiment | 20 | 6 | 1 | / | Yes |
| In vivo experiment |  | 5 | 1 | / | Yes |
| Metabolomics analysis |  | 6 | 1 | / | Yes |
| Proteomics analysis |  | 3 | 1 | / | Yes |
| Immunofluorescence microscopy |  |  |  | 3 | Yes |
| TEM analysis |  |  |  |  | Yes |
| SEM analysis |  |  |  |  | Yes |

S-Table 2. PLT(DMSO-) *vs* PLT(DMSO+) differential metabolites

| **name** | **Vipscore** | **log2FC** | **pValue** | **FDR** | **significance** |
| --- | --- | --- | --- | --- | --- |
| Dopamine | 0.285714 | 1.126389 | 0.046802 | 0.208378 | Up |
| N-Methylnicotinamide | 0.1732 | -0.64459 | 0.006862 | 0.061108 | Down |
| L-Alloisoleucine | 0.21158 | -0.75067 | 0.006125 | 0.061108 | Down |
| Phenylacetic acid | 0.236757 | -0.93003 | 0.008076 | 0.067052 | Down |
| L-Norleucine | 0.262881 | -1.00413 | 0.000706 | 0.032985 | Down |
| L-Tryptophan | 0.281963 | -1.06639 | 0.004716 | 0.057287 | Down |
| Leucine | 0.290841 | -1.11546 | 0.012922 | 0.088134 | Down |
| Phenylalanine | 0.293394 | -1.03029 | 0.000883 | 0.033036 | Down |
| Norvaline | 0.300294 | -1.11931 | 0.004411 | 0.057287 | Down |
| Isoleucine | 0.30908 | -1.18703 | 0.011467 | 0.085777 | Down |
| Glutaric acid | 0.314384 | -1.23684 | 0.002768 | 0.047064 | Down |
| Inosinic acid | 0.324505 | -1.24274 | 0.015227 | 0.094913 | Down |
| S-Adenosylhomocysteine | 0.361634 | -1.3554 | 0.003668 | 0.057154 | Down |
| Methionine | 0.378754 | -1.5066 | 0.039949 | 0.188109 | Down |
| Niacinamide | 0.391405 | -1.48269 | 0.010187 | 0.079373 | Down |
| L-Acetylcarnitine | 0.419657 | -1.53174 | 0.015913 | 0.095994 | Down |
| Inosine | 0.424907 | -1.48942 | 0.002713 | 0.047064 | Down |
| Leucylalanine | 0.432553 | -1.49155 | 0.004992 | 0.057287 | Down |
| L-Valine | 0.467861 | -1.82472 | 0.01834 | 0.104612 | Down |
| Guanosine monophosphate | 0.543678 | -1.84459 | 0.000232 | 0.028881 | Down |
| L-Asparagine | 0.553913 | -1.95512 | 0.000316 | 0.028881 | Down |
| 4-hydroxy-L-proline | 0.5664 | -2.00451 | 0.002519 | 0.047064 | Down |
| Benzoic acid | 0.599058 | -1.37354 | 0.040995 | 0.188109 | Down |
| Malic acid | 0.60369 | -2.23739 | 0.013196 | 0.088134 | Down |
| L-Tyrosine | 0.647301 | -1.95636 | 0.013034 | 0.088134 | Down |
| Cytidine monophosphate | 0.737932 | -2.62296 | 0.002178 | 0.047064 | Down |
| Pipecolic acid | 0.739357 | -2.62668 | 0.008247 | 0.067052 | Down |
| Pyroglutamic acid | 0.77269 | -2.79765 | 0.006692 | 0.061108 | Down |
| Methylcysteine | 0.780515 | -2.15895 | 0.020558 | 0.113069 | Down |
| Ribonolactone | 0.782286 | -2.46353 | 0.002597 | 0.047064 | Down |
| L-Arginine | 1.246166 | -2.20751 | 0.040473 | 0.188109 | Down |
| Myristic acid | 1.40888 | -2.5314 | 0.005208 | 0.057287 | Down |
| Asymmetric dimethylarginine | 1.432148 | -2.05902 | 0.021905 | 0.117037 | Down |
| Glutathione | 1.560333 | -3.23689 | 0.018461 | 0.104612 | Down |
| 3-Pyridylacetic acid | 2.077117 | -1.91815 | 0.0065 | 0.061108 | Down |
| Taurine | 2.096594 | -9.14272 | 0.041243 | 0.188109 | Down |
| Xanthosine | 2.129642 | -9.79903 | 0.036009 | 0.181992 | Down |
| Lactic acid | 2.170756 | -3.36064 | 0.001719 | 0.047064 | Down |
| Uridine | 2.743371 | -11.1286 | 0.025625 | 0.133106 | Down |
| Indoleacetic acid | 2.778811 | -7.22269 | 0.000463 | 0.028881 | Down |
| Proline | 2.92606 | -3.96933 | 0.004493 | 0.057287 | Down |
| Linoelaidic acid | 3.298027 | -3.73311 | 0.013738 | 0.088586 | Down |
